# Addressing physical, functional, and physiological outcomes in older adults via integrated mHealth intervention

**DOI:** 10.1093/geroni/igab046.3356

**Published:** 2021-12-17

**Authors:** Melba Hernandez-Tejada, Sundaravadivel Balasubramanian, John Bian, Mohan Madisetti, Alexis Nagel, Samantha Bernstein, Teresa Kelechi

**Affiliations:** 1 University of Texas HSC at Houston, Houston, Texas, United States; 2 Medical University of South Carolina, Charleston, South Carolina, United States

## Abstract

Objective: We evaluated components of an integrated mobile (m)Health-based intervention "Activate for Life" (AFL) on health outcomes in lower-income older adults (65 years and older). Method: AFL incorporates balance (Otago; OG), physical strength (Gentle Yoga and Yogic Breathing; GYYB), and mental engagement (Behavioral Activation; BA) components. Thirty participants were randomly allocated to one of three Arms (n=10 per each arm): OG (Arm 1), (OG+GYYB (Arm2), or OG+GYYB+BA (Arm 3, or full AFL). Groups were evaluated for physical, functional and physiological endpoints at baseline, and posttreatment (12-weeks and/or 3-month follow up). Results: Improvements over time in pain interference and 1,5 Ag biomarker were noted for all groups. No significant changes were observed in other physical, functional and physiological measures. DiscussionThis study illustrated potential benefits of the AFL intervention on the health of lower-income older adults and lessons learned from this pilot will be used to make improvements for a large-scale randomized controlled trial.

